# Inelastic Triatom-Atom Quantum Close-Coupling Dynamics in Full Dimensionality: All Rovibrational Mode Quenching of Water Due to the H Impact on a Six-Dimensional Potential Energy Surface

**DOI:** 10.1021/acs.jpclett.4c02865

**Published:** 2024-11-04

**Authors:** Benhui Yang, Chen Qu, J. M. Bowman, Dongzheng Yang, Hua Guo, N. Balakrishnan, R. C. Forrey, P. C. Stancil

**Affiliations:** † Department of Physics and Astronomy and Center for Simulational Physics, University of Georgia, Athens, Georgia 30602, United States; ‡ Department of Chemistry, Emory University, Atlanta, Georgia 30322, United States; ¶ Department of Chemistry and Chemical Biology, Center for Computational Chemistry, University of New Mexico, Albuquerque, New Mexico 87131, United States; § Department of Chemistry and Chemical Biology, Center for Computational Chemistry, University of New Mexico, Albuquerque, New Mexico 87131, United States; ∥ Department of Chemistry and Biochemistry, University of Nevada, Las Vegas, Nevada 89154, United States; ⊥ Department of Physics, Penn State University, Berks Campus, Reading, Pennsylvania 19610, United States

## Abstract

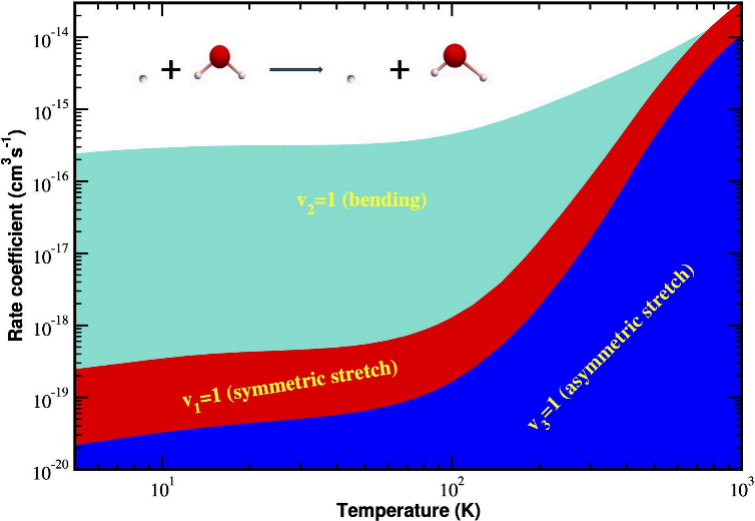

The rovibrational level populations, and subsequent emission in various astrophysical environments, are driven by inelastic collision processes. The available rovibrational rate coefficients for water have been calculated using a number of approximations. We present a numerically exact calculation for the rovibrational quenching for all water vibrational modes due to collisions with atomic hydrogen. The scattering theory implements a quantum close-coupling (CC) method on a high level ab initio six-dimensional (6D) potential energy surface (PES). Total rovibrational quenching cross sections for excited bending levels were compared with earlier results on a 4D PES with the rigid-bender close-coupling (RBCC) approximation. General agreement between 6D-CC and 4D-RBCC calculations are found, but differences are evident including the energy and amplitude of low-energy orbiting resonances. Quenching cross sections from the symmetric and asymmetric stretch modes are provided for the first time. The current 6D-CC calculation provides accurate inelastic data needed for astrophysical modeling.

Water is one of the most abundant and ubiquitous species in warm regions of the interstellar medium (ISM) with a rich spectrum of rotational and rovibrational transitions. Since the detection of H_2_O maser 6_16_-5_23_ rotational transition near 22 GHz in 1969,^1^ water has been widely observed in a variety of diverse astrophysical environments.^2−7^ Water was the focus of a key program for the Herschel Space Observatory, Water In Star-forming regions with Herschel (WISH).^8^ The Mid-Infrared Instrument (MIRI) on-board the James Webb Space Telescope (JWST) can probe hot and warm water in shocks and inner disk surface layers through the 6 μm vibration–rotation band and pure rotational lines for λ > 10 μm. The ground-based telescopes VLT-CRIRES+ and Keck-NIRSPEC, and in the future ELT-METIS, can also spectrally resolve H_2_O rovibrational emission lines at 3 μm in star- and planet-forming disks at radii of ∼100 AU and constrain their location through systematic velocity moment maps. Moreover, ELT-METIS can spatially resolve the lines down to a few AU and distinguish a disk surface layer from a disk wind. Vibrational transitions of H_2_O were also detected using the EXES spectrometer on the Stratospheric Observatory for Far Infrared Astronomy (SOFIA).^9,10^ Baudry et al.^11^ reported the observation of ten rotational transitions in the ground and excited vibrational states up to (*v*_1_, *v*_2_, *v*_3_) = (0, 1, 1) of H_2_O in the ATOMIUM Band 6 survey with Atacama Large Millimeter/submillimeter Array (ALMA). For chemical abundance modeling, they made the following remark: “ ··· an in-depth development of H_2_O line excitation models awaits newer collision rates and needs to incorporate higher vibrational states, hopefully up to the (0, 3, 0), (1, 1, 0) and (0, 1, 1) states, together with line overlap effects between para- and ortho-water.” Moreover, water plays a key role in the physical and chemical evolution and formation of the inner regions of protoplanetary disks (PPDs). JWST provides opportunities to investigate the chemical properties of the warm inner regions of disks, and the spectral resolution of the JWST/MIRI spectrometer is essential to identifying H_2_O and accurately determining its column density and temperature.^12^ Recently, Perotti et al.^13^ reported JWST observations of PDS 70 and the MIRI spectrum showed water lines in the 7 μm spectral window, where the rovibrational transitions of the bending mode of H_2_O was modeled assuming a thermal population of rovibrational levels. Further, the inner regions of the T Tauri star Sz 98 was examined using JWST/MIRI spectra.^14^ Both rovibrational and pure rotational emission of H_2_O were detected in the emitting layers.

The water molecule is a typical asymmetric top with the three vibrational modes of H_2_O designated (*v*_1_, *v*_2_, *v*_3_). *v*_*i*_ represent the vibrational quanta of normal modes for symmetric stretch, bend, and asymmetric stretch, respectively, with the vibrational ground state designated as (0, 0, 0). The rotational states of H_2_O are labeled by *j*_*K*_*a*_,*K*_*c*__, where *j* is the total rotational angular momentum, *K*_*a*_ and *K*_*c*_ are the projections of *j* on the molecule-fixed *a* and *c* axes of H_2_O, respectively. Water has two nuclear-spin isomers, para (*I* = 0, singlet) and ortho (*I* = 1, triplet), determined by the overall spin of the two hydrogen nuclei (*I*_H_ = 1/2). Para-H_2_O exists in rovibrational states with even *K*_*a*_ + *K*_*c*_ + *v*_3_ and ortho-H_2_O exists with odd *K*_*a*_ + *K*_*c*_ + *v*_3_.^15^

For the nonreactive collision of the water molecule with atomic hydrogen, a number of potential energy surfaces (PESs)^16−19^ have been developed. Zhang et al.^16^ reported the first three-dimensional (3D) ab initio potential for the rigid water-atomic hydrogen system based on Møller–Plesset fourth-order perturbation theory and Dagdigian and Alexander^17^ developed another 3D PES using the restricted coupled-cluster single, double, and perturbative triple (RCCSD(T)) method. H_2_O was treated as a rigid-rotor, with bond length and bond angle (*r* = 1.8361 bohr and γ = 104.69°) corresponding to the vibrationally averaged geometry. A new 3D PES of H_2_O-H was constructed by McCarver and Hinde, based on CCSD(T) calculations. In their work, H_2_O was treated as a rigid-monomer held at its experimentally determined equilibrium geometry. Very recently, a four-dimensional (4D) PES including the H_2_O bending mode was reported by Cabrera-González et al.^19^ The ab initio calculation was carried out using the unrestricted CCSD(T)-F12a (UCCSD(T)-F12a) level of theory, but with the OH bond length fixed at 0.957 Å.

The quantum theory for scattering of a rigid asymmetric top, such as H_2_O, with a spherical atom was developed in 1976 by Garrison et al.^20^ and Green.^21^ Using the 3D PES of Dagdigian and Alexander^17^ and close-coupling formalism, Daniel et al.^22^ calculated rotationally inelastic rate coefficients for the first 45 levels of H_2_O and for temperatures in the range of 5–1500 K. For the rovibrational excitation of H_2_O in collision of H, Bissonneette and Clary^23^ performed close-coupling and coupled states approximation calculations of rovibrational energy transfer of H_2_O due to H collisions. Cross sections were obtained for excitation of the asymmetric stretching vibration *v*_3_ = 1 involving *v*_1_ ↔ *v*_3_ transitions. A quantum study of the bending relaxation of H_2_O due to H was presented by Cabrera-González et al.^19^ using the rigid-bender-close-coupling (RBCC) method. The RBCC method was also used in the study of H_2_O in collision with H_2_.^24^ More recently, a full-dimensional inelastic scattering code for triatom–atom collisions, ABC+D, was reported by Yang et al.,^25^ based on the time-independent quantum mechanical coupled channel method. The ABC+D code has been successfully used to study rovibrational scattering between H_2_O and Cl, He, and Ar atoms.^26−28^

We report a new 6D, full-dimensional PES for H_2_O–H and the first coupled-channel (CC), full-dimensional scattering calculation with full angular momentum coupling for rovibrational transitions of water due to H impact for the first excited states of all vibrational modes. The calculations were performed with the ABC+D code and an invariant polynomial fit of this new 6D PES. Here we briefly describe the methods used in rovibrational inelastic scattering calculations and PES calculation and fit. The reader is referred to refs (26) and (29) for additional details.

The total Hamiltonian for the collision of a triatomic molecule ABC and a spherical atom in the coordinate system presented by Brocks, van der Avoird, Sutcliffe, and Tennyson (BAST)^25,30^ can be expressed as (in atomic units),
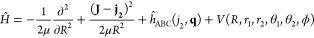
1where μ is the reduced mass for the collision system and **q** ≡ (*r*_1_, *r*_2_, θ_1_) are intramolecular Radau coordinates for the triatom ABC. The collision coordinate from the center of mass of ABC to the atom is described in Jacobi coordinates as (*R*, θ_2_, ϕ). *V* (*R*, *r*_1_, *r*_2_, θ_1_, θ_2_, ϕ) is the interaction potential between ABC and atom D. **j**_**2**_ is the rotational angular momentum of ABC and **J** the total angular momentum. *ĥ*_ABC_ is the Hamiltonian for ABC with the parity-adapted eigenfunctions of ABC obtained by solving the equation,
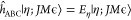
2where channel index η is defined as η ≡ (*j*_2_*tK*). *t* is a rovibrational state of ABC, *E*_η_ is the internal energy of the ηth channel, and ϵ is the system parity. *K* and *M* are the projections of *J* onto the body-fixed and space-fixed *z* axes, respectively. The total wave function of the scattering system can be expanded in terms of eigenfunctions of the triatomic molecule:
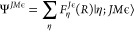
3The resulting second-order CC differential equations for the radial functions are expressed as^31^
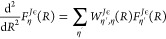
4The coupling matrix *W*_*η′*, η_^*Jϵ*^(*R*) is given by
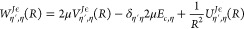
5where *E*_*c*,η_ is the collision energy. The centrifugal matrix **U** is expressed as
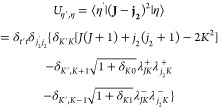
6where . The matrix elements of the coupling potential, **V**, are,
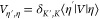
7The log-derivative method^31,32^ was applied to solve the CC eq 4. Within the CC formulation, the state-to-state integral cross section for a transition from an initial state *j*_2_*t* to a final state *j*_2_*′t′* is given by
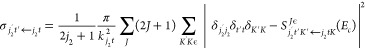
8where the wave vector  and *S^Jϵ^*_*j*_2_*′t′K′* ← *j*_2_*tK*_ is the *S* matrix.

The interaction energy between H and H_2_O was computed using MOLPRO2010^33^ at the RCCSD(T)-F12b^34,35^ level of theory with the aug-cc-pVQZ basis. The basis set superposition error was removed by applying the counter-poise correction. The data set consists of 151,027 configurations and corresponding energies. The geometries in the data set were sampled on a 6D grid. This includes 20 values for *R* ranging from 3.5 to 30.0 bohr, 6 different values for θ over [0, π/2], and 7 different ϕ values over [0,π].

From the full data set, 75,476 points with *R* ≥ 8.0 bohr were selected as the long-range data. The final PES combines a fit to the full data set (denoted as *V*_I_) and a fit to the long-range data (denoted as *V*_II_). The potential energy of the combined PES, referred to as VHH2O, is given by
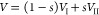
9where *s* is a switching function defined as,
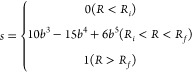
10where *R*_*i*_ = 8.0 *a*_0_ and *R*_*f*_ = 10.0 *a*_0_, and *b* = (*R* – *R*_*i*_)/(*R*_*f*_ – *R*_*i*_). Both *V*_I_ and *V*_II_ have been fitted in 6D using the permutation invariant polynomial method via monomial symmetrization.^36^ Both fits for *V*_I_ and *V*_II_ used a maximum polynomial order of 8, with 1589 linear coefficients. The root-mean-square (rms) fitting error of *V*_I_ is 2.93 cm^–1^, while the rms of *V*_II_ is 0.025 cm^–1^. The PES is smooth and free of bumps or holes.

Figure 1 shows a two-dimensional contour plot around the global minimum of θ and ϕ for the rigid rotor H_2_O–H potentials of the 6D PES for H–H_2_O distance *R* = 6.5 bohr. Note that *R*, θ, ϕ are body-fixed polar coordinates defined in Figure 1 of ref (17)., and *R* is the distance between H and center of mass of H_2_O. In Table S1, the well depth and equilibrium geometry of the previously reported H_2_O-H PESs are presented. To further assess the accuracy of the 6D PES, we performed rigid-rotor inelastic scattering calculations as described in Section S2 in the Supporting Information. The good agreement between our rotational de-excitation rate coefficients and the results obtained with a 3D PES^22^ confirms the accuracy of our 6D PES.

**Figure 1:**
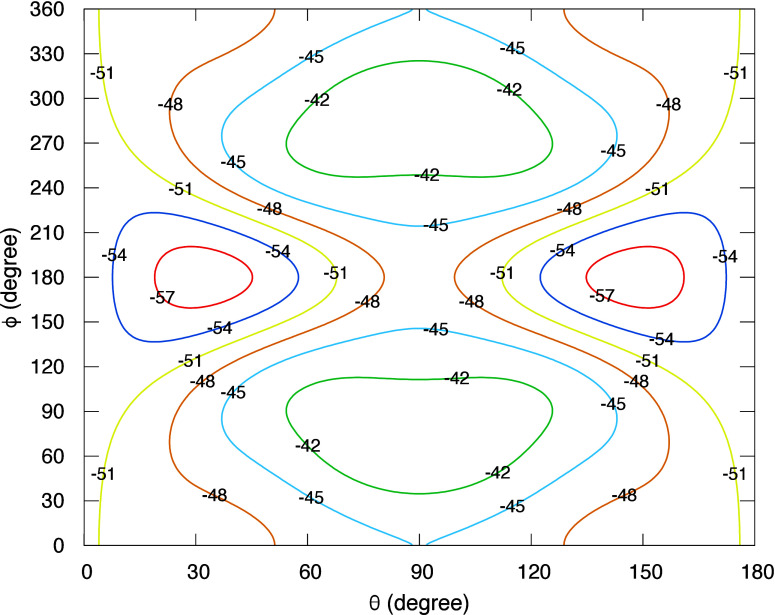
Contour plot of PES as a function of orientation angles θ and ϕ, *R* = 6.5 bohr. H_2_O is treated as a rigid rotor at the vibrationally averaged geometry. *R*, θ, ϕ are body-fixed polar coordinates defined in Figure 1 of ref (17). The H_2_O molecule lies in the *xz*-plane with the origin centered on the H_2_O center of mass with *z* axis oriented along the A inertial axis of H_2_O. The unit of potential energy is cm^–1^.

Full-dimensional rovibrational scattering calculations were carried out using the ABC-D code^25^ in which the CC equations were propagated from R = 3.5 to 30.0 *a*_0_. The number of Gauss-Legendre quadrature points adopted for θ_1_, θ_2_, and ϕ to integrate out the angular dependencies in the matrix elements were *N*_θ_1__ = 23, *N*_θ_2__ = 19, and *N*_ϕ_ = 20, respectively. The number of potential optimized discrete variable representation (PODVR)^37^ points were chosen as *N*_*r*1_ = *N*_*r*2_ = 4. The energy truncation for the contracted basis functions employed was *E*_η_^*max*^ = 6000 cm^–1^. The log-derivative propagation was carried out with different step sizes Δ*R* in different ranges of *R*: Δ*R* = 0.04 *a*_0_ in *R* ∈ [3.5, 5.0] *a*_0_, Δ*R* = 0.08 *a*_0_ in *R* ∈ [5.0, 10.0] *a*_0_, and Δ*R* = 0.1 *a*_0_ in *R* ∈ [10.0, 30.0] *a*_0_. Furthermore, convergence tests of the cross sections with respect to partial wave summation and rovibrational basis have been performed. The maximum number of partial waves *J* in the scattering calculations is up to 100. All these parameters yielded results converged to within 5%. We focus first on the vibrational quenching from the first excited state of the water bending mode (*v*_2_ = 1), (010). The calculation of rovibrational state-to-state quenching cross sections and rate coefficients was performed for initial rotational states of para-H_2_O: 0_00_, 1_11_, 2_02_, 2_11_, 3_13_, and 2_20_, and of ortho-H_2_O: 1_01_, 1_10_, 2_12_, 3_03_, and 2_21_.

Figure 2 shows the distribution of final rotational states of para-H_2_O in the ground vibrational state (000) following de-excitation from initial state (010)0_00_ at collision energies of 1.0 and 100 cm^–1^. The final rotational distribution is broad and centered in the region of low *j* levels. The distributions extend up to higher rotational levels for both collision energies. No propensity rules are evident, though the dominant transition is for Δ*j* = 2, Δ*K*_*a*_ = 0, and Δ*K*_*c*_ = 2. The weakest transitions typically have Δ*j* ≠ Δ*K*_*a*_ and/or Δ*j* ≠ Δ*K*_*c*_. Some final states are missing in the plot because of their small cross section magnitudes. In Figure S3 we also show the distribution of final rotational states but for ortho-H_2_O in the ground vibrational state. The distribution patterns are similar to para-H_2_O.

**Figure 2:**
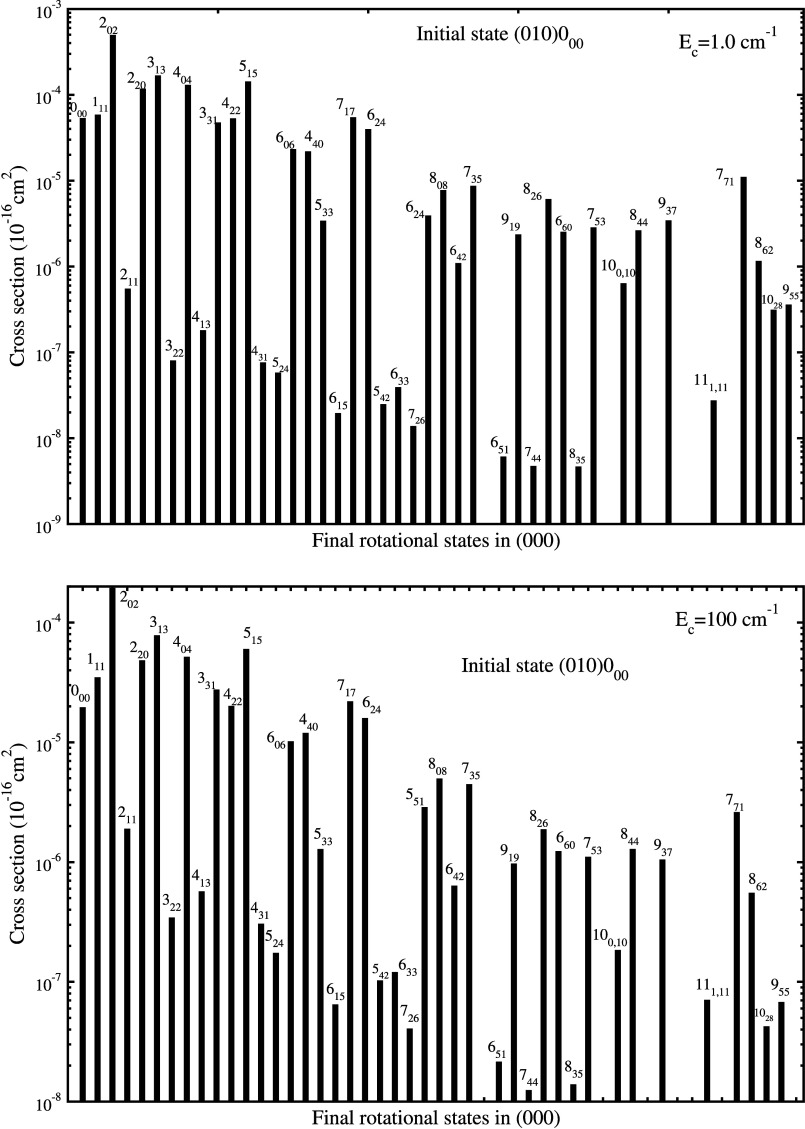
Rotational state distribution of para-H_2_O in (000) from initial state (010)0_00_ in collision with H. The final rotational states are energy ordered. Upper: collision energy = 1.0 cm^–1^; lower: collision energy = 100.0 cm^–1^.

In Figure 3 we present the state-to-state cross section for rovibrational quenching from H_2_O vibrational states (010), (100), and (001). Because of a large number of final states, as examples, we only present some selected final states for each initial state. Figure 3(a) shows the results from the initial state (010)2_20_ for para-H_2_O with H. It can be seen that two groups of cross sections are shown for Δ*v*_2_ = −1 (lower) and Δ*v*_2_ = 0 (upper). Resonance structures are present for energies between ∼1 and ∼100 cm^–1^. The results of ortho-H_2_O with H shown in Figure S4 give similar behavior as para-H_2_O. The state-to-state rate coefficients for the vibrational quenching (010) → (000) corresponding to the cross sections shown in Figure 3(a) and Figure S4 are presented in Figure S5 for temperatures ranging from 5 to 1000 K. The rate coefficients display similar trends as shown in Figure 3 (a) and Figure S4 for the cross section.

**Figure 3:**
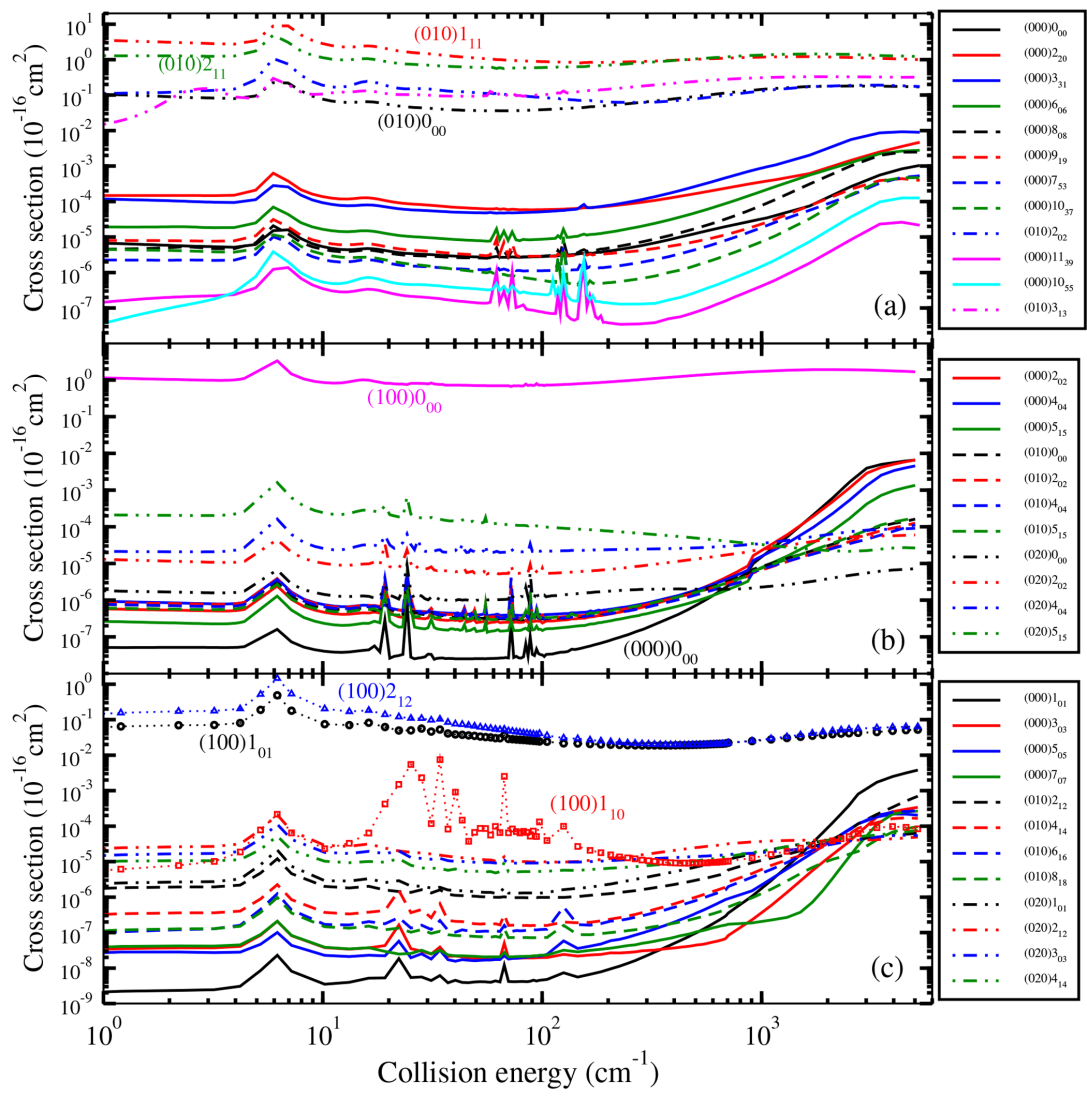
State-to-state rovibrational quenching cross sections of H_2_O in collision with H. (a): from initial para-H_2_O state (010)2_20_; (b): from initial para-H_2_O state (100)1_11_; (c): from initial ortho-H_2_O state (001)0_00_.

We have also extended the full-dimensional scattering to the rovibrational quenching from the symmetric stretching vibrational state (100) of para-H_2_O and asymmetric stretching vibration (001) of ortho-H_2_O. We believe that cross sections from these vibrational modes of water are computed here for the first time. The cross sections are displayed in Figure 3 (b) and Figure 3(c) for selected final rotational states in vibrational states (000), (010), (020), and (100). As the symmetric and antisymmetric vibrational modes are higher in energy, exoergic transitions to excited bending modes (010) and (020) are accessible as shown in the figures. All the vibrational quenching cross sections display resonances in the low collisional energy region due to quasibound states supported by the attractive part of the interaction potential. To identify the partial wave contribution to the resonances at collision energies near 5 to 8 cm^–1^, we show in Figure S6 the *J*-resolved contributions to the quenching cross sections to selected final states from initial state (010)2_20_. The dominant partial waves are *J* = 6, 5, 2, and 5 for final states (000)0_00_, (000)2_20_, (010)0_00_, and (010)2_02_, respectively.

The total vibrational quenching cross section from an initial state (010)*j*_*K*_*a*_ *K*_*c*__ can be obtained by summing the state-to-state cross sections over all final rotational states *j'*_*K*_*a*_*′ K*_*c*_*′*_ in (000). Figure 4 displays the total vibrational quenching cross sections of para-H_2_O from initial states (010) *j*_*K*_*a*_,*K*_*c*__ = 0_00_, 1_11_, 2_02_, 2_11_, 2_20_, and 3_13_. The upper panel shows the comparison between our total quenching cross section from initial state (010)0_00_ with the RBCC result of Cabrera-González et al.^19^ In general, the agreement is reasonable, though the current cross section is somewhat smaller. The two dominant resonances are reproduced in the current calculations, but additional resonances over the collision energy range of 30 to 200 cm^–1^ are found due to the finer energy grid adopted here. The differences can be attributed to the features of the PESs used in the scattering calculations, in particular, the different well depths of the PESs as shown in Table S1. The lower panel of Figure 4 shows the total vibrational (010) to (000) quenching cross section from initial states *j*_*K*_*a*_,*K*_*c*__ = 0_00_, 1_11_, 2_02_, 2_11_, 2_20_, and 3_13_. All of the total quenching cross sections show similar behavior and reflect the presence of a large number of resonances in the intermediate energy region between ∼3 and ∼200 cm^–1^. For energies larger than 200 cm^–1^, the cross sections increase with increasing collision energy, typical of vibrational–rotational/translational energy transfer. Figure S7 displays the total quenching cross sections for ortho-H_2_O. Our 6D-CC calculations show good agreement with the 4D-RBCC results, but we again find additional resonances. The total quenching cross sections from initial states (010) *j*_*K*_*a*_,*K*_*c*__ = 1_01_, 1_10_, 2_12_, 2_21_, and 3_03_ are also shown in Figure S7.

**Figure 4:**
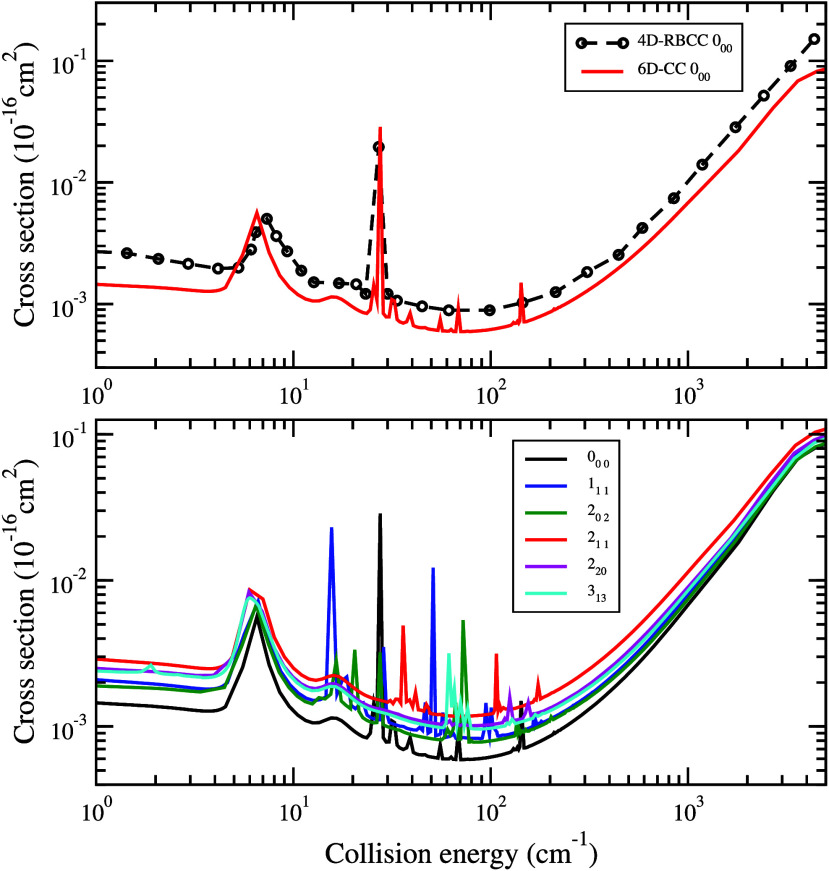
Total quenching cross section from (010)*j*_*K*_*a*_,*K*_*c*__ to (000) of para-H_2_O in collision with H. (Upper panel) Comparison between present 6D-CC calculation with the 4D-RBCC results of Cabrera-González et al.^19^ from initial state (010)0_00_. (Lower panel) Present results from initial state *j*_*Ka*,*Kc*_ = 0_00_, 1_11_, 2_02_, 2_11_, 2_20_, and 3_13_.

Finally in Figure 5 we compare the state-to-state quenching cross section from initial states (010)0_00_, (100)1_11_, and (001)0_00_ to the lowest rotational states in the ground vibrational state (000) of H_2_O. At collision energies below ∼1500 cm^–1^, the transition from bending vibrational state (010)0_00_, with smaller energy gap between initial and final states, displays a much larger cross section than from the initial vibrational stretching states which have similar energy levels.

**Figure 5:**
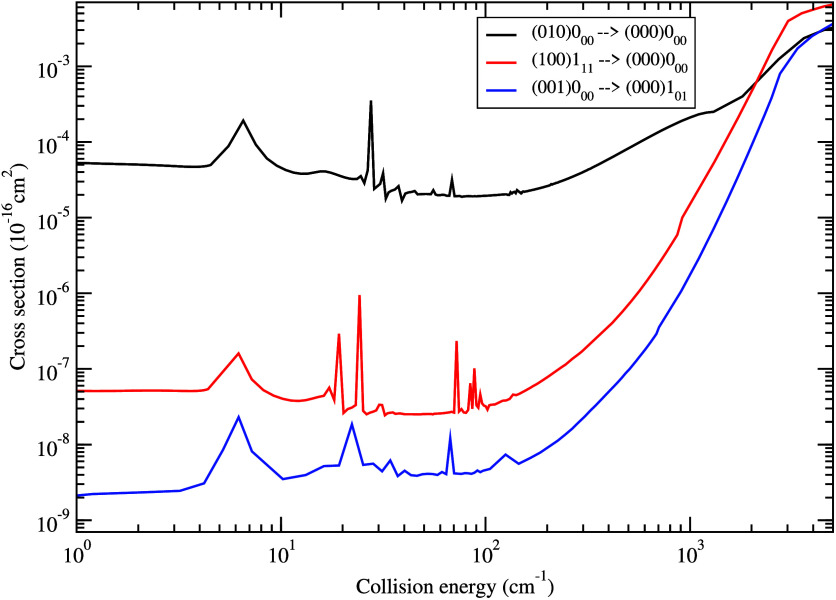
State-to-state quenching cross section from initial states (010)0_00_, (100)1_11_, (001)0_00_ to the lowest rotational states in the ground vibrational state (000) of H_2_O in collision with H.

Calculations of quenching from higher excited rotational and vibrational states of H_2_O are in progress. The current results, future large-scale coupled-states (CS) approximation calculations, and machine-learning methods will be essential in the construction of a database of H_2_O vibrational and rotational quenching rate coefficients urgently needed for astrophysical modeling.

## Supporting Information Available

A table comparing H_2_O-H PES global minimum, rigid-rotor scattering results, and additional rovibrational scattering cross sections and rate coefficients of H_2_O in collision with H (PDF)
Transparent Peer Review report available (PDF)
