# Supplementary material for: Inelastic Triatom-Atom Quantum Close-Coupling Dynamics in Full Dimensionality: All Rovibrational Mode Quenching of Water Due to the H Impact on a Six-Dimensional Potential Energy Surface
Source: J Phys Chem Lett. 2024 Nov 4;15(45):11312–9. doi: 10.1021/acs.jpclett.4c02865 (PMC11571227; doi:10.1021/acs.jpclett.4c02865)
Supplement: Supplementary file 1 — jz4c02865_si_001.pdf [file jz4c02865_si_001.pdf]

Supporting Information: Inelastic Triatom-Atom Quantum Close-Coupling Dynamics in Full Dimensionality: all rovibrational mode quenching of water due to H impact on a six-dimensional potential energy surface

Benhui Yang,[*,†] Chen Qu,[‡] J. M. Bowman,[‡] Dongzheng Yang,[¶] Hua Guo,[¶] N. Balakrishnan,[§] R. C. Forrey,[‖] and P. C. Stancil[*,†]

†*Department of Physics and Astronomy and Center for Simulational Physics, University of Georgia, Athens, GA 30602, United States*

‡*Department of Chemistry, Emory University, Atlanta, GA 30322, United States*

¶*Department of Chemistry and Chemical Biology, Center for Computational Chemistry, University of New Mexico,Albuquerque, NM 87131, United States*

§*Department of Chemistry and Biochemistry, University of Nevada, Las Vegas, NV 89154, United States*

‖*Department of Physics, Penn State University, Berks Campus, Reading, PA 19610, United States*

E-mail: byang@uga.edu; pstancil@uga.edu

S1. The Potential Energy Surface

In Table S1, the well depth and equilibrium geometry of the published H_2O-H PESs are presented.

Table S1: Minimum energies and equilibrium geometries of H_2O-H PESs.

PES	R (bohr)	θ_2 (degrees)	ϕ (degrees)	$V_{\rm min}$ (cm^{-1})
VHH2O	6.53	123.5	0	-57.8
Cabrera-González et al.[1]	6.44	122.53	0	-59.58
Dagdigian & Alexander[2]	6.44	119.04	0	-61.0
McCarver & Hinde[3]	6.44	122.25	0	-61.3

S2. Rigid-rotor scattering calculation

The state-to-state rate coefficients from initial state i to final state j at a temperature T can be obtained by thermally averaging the corresponding state-to-state integral cross sections over a Maxwellian kinetic energy distribution,

$$k_{j\leftarrow i}(T) = \left(\frac{8}{\pi\mu\beta}\right)^{1/2} \beta^2 \int_0^\infty E_c \sigma_{j\leftarrow i}(E_c) \exp(-\beta E_c) dE_c, \tag{S.1}$$

where $\beta = (k_{\rm B}T)^{-1}$, $k_{\rm B}$ is Boltzmann's constant, E_c is the collision energy, and μ is the reduced mass of the system.

Using the scattering code Molscat[4] with the VHH2O PES, we calculated state-to-state cross sections and rate coefficients for rotational de-excitation of H_2O in the ground vibrational state in collisions with H. H_2O was held rigid with its three normal coordinates held at their equilibrium positions. Rotational energy levels of H_2O were taken from Kyrö[5] and the scattering calculations considered the initial states 1_{11} and 5_{15} for para-H_2O, and 1_{10} and 5_{05} for ortho-H_2O. Cross sections were computed for collision energies ranging from 1 to 5000 cm^{-1}.

In Fig. S1 the state-to-state quenching rate coefficients are presented for the quenching transitions $1_{1,1} \to 0_{0,0}$ and $1_{1,0} \to 1_{0,1}$. Our results are in excellent agreement with the calculations of Daniel et al.[6] Fig. S2 makes a similar comparison of our state-to-state rate coefficients for initial para-H_2O state $5_{1,5}$ (Upper panel) and ortho-H_2O state $5_{0,5}$ (Lower panel). Generally, for both

para- and ortho-H_2O the agreement is good, in particular at temperatures above 200 K. For some transitions, the small discrepancies are likely due to differences in the adopted PESs.

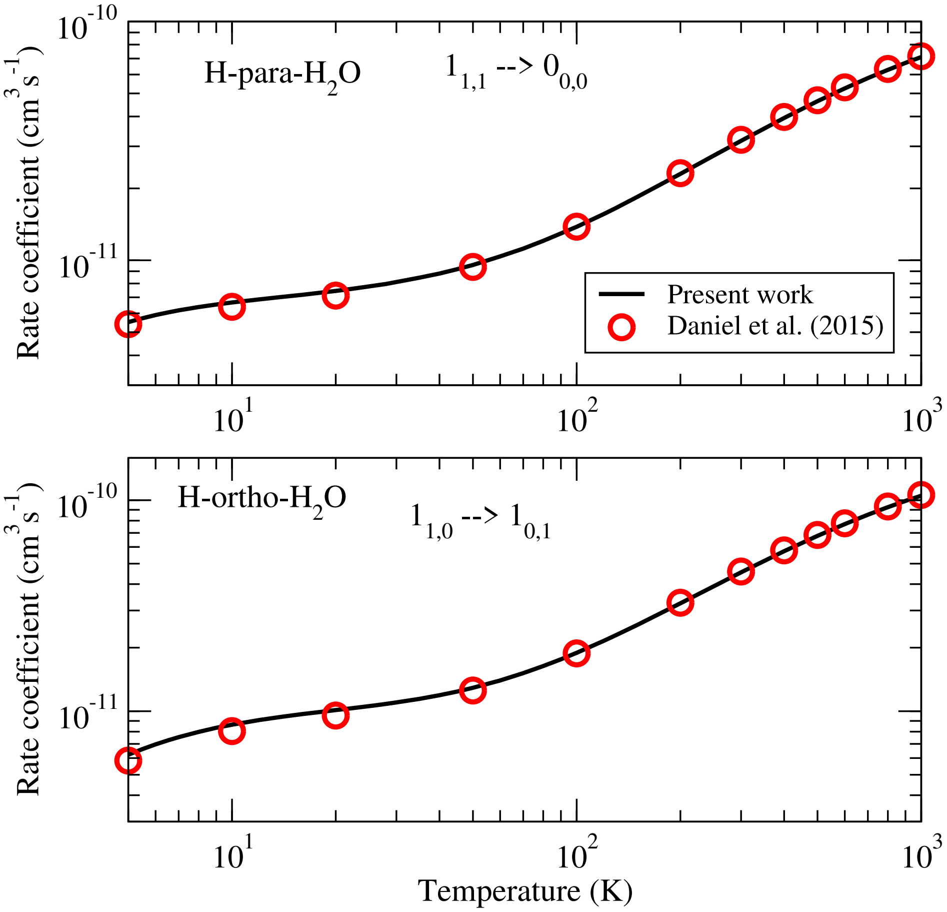

Figure S1: Rotational state-to-state de-excitation rate coefficients of H_2O in ground vibrational state in collision with H. (Upper) Para-H_2O, transition $1_{1,1} \rightarrow 0_{0,0}$, (Lower) ortho-$H_2O$, transition $1_{1,0} \rightarrow 1_{0,1}$.

S3. Full-dimensional rovibrational scattering calculations

In Fig. S3, we show the distribution of final rotational states but for ortho-H_2O in the ground vibrational state (000). The distribution patterns are similar to para-H_2O.

Fig. S4 shows the state-to-state quenching cross section from initial state $(010)2_{21}$ to some selected rotational states in vibrational state (000) and (010).

The state-to-state rate coefficients for the vibrational quenching $(010) \rightarrow (000)$ are presented in Fig. S5 for temperatures ranging from 5 to 1000 K.

To identify the partial wave contribution to the resonances at low collision energies, in particular, at collision energies near 5 to 8 cm^{-1}, we show in Fig. S6 the J-resolved contributions to the quenching cross sections to final states (000)0_{00}, (000)2_{20}, (010)0_{00}, and (010)2_{02}. The initial state is (010)2_{20} and collision energies are between 3.5 and 20 cm^{-1}. It can be seen that between collision energies 5 to 8 cm^{-1} the dominant partial waves are J =6, 5, 2, and 5 for final states (000)0_{00}, (000)2_{20}, (010)0_{00}, and (010)2_{02}, respectively.

Fig. S7 displays the total quenching cross sections for ortho-H_2O. The upper panel shows comparison between our total quenching cross sections from (010)2_{21} and (010)3_{03} with the 4D-RBCC result of Cabrera-González et al.[1] Our 6D-CC calculations show good agreement with the 4D-RBCC results, but we again find additional resonances. The total quenching cross sections from initial states (010) j_{K_a,K_c}=1_{01}, 1_{10}, 2_{12}, 2_{21}, and 3_{03} are shown in the lower panel.

References

(1) Cabrera-González, L. D.; Denis-Alpizar, O.; Páez-Heernández, D.; Stoecklin, T. Quantum study of the bending relaxation of H_2O by collision with H. *Monthly Notices of the Royal Astronomical Society.* **2022**, *514,* 4426-4432.

(2) Dagdigian, P. J.; Alexander, M. H. Exact quantum scattering calculations of transport properties for the H_2O-H system. *J. Chem. Phys.* **2013**, *139*, 194309.

(3) McCarver, G. A.; Hinde, R. J. High accuracy ab initio potential energy surface for the H_2O-H van der Waals dimer. *J. Chem. Phys.* **2021**, *155*, 114302.

(4) Hutson, J. M.; Green, S. MOLSCAT computer code, version 14. **1994**.

(5) Kyrö, E. Centrifugal distortion analysis of pure rotational spectra of $H_2^{16}O$, $H_2^{17}O$, and $H_2^{18}O$. *J. Mol. Spectrosc.* **1981**, *88*, 167.

(6) Daniel, F.; Faure, A.; Dagdigian, P. J.; Dubernet, M.-L.; Lique, F.; des Forets, G. P. Collisional excitation of water by hydrogen atoms. *Monthly Notices of the Royal Astronomical Society.* **2015**, *446*, 2312-2316.

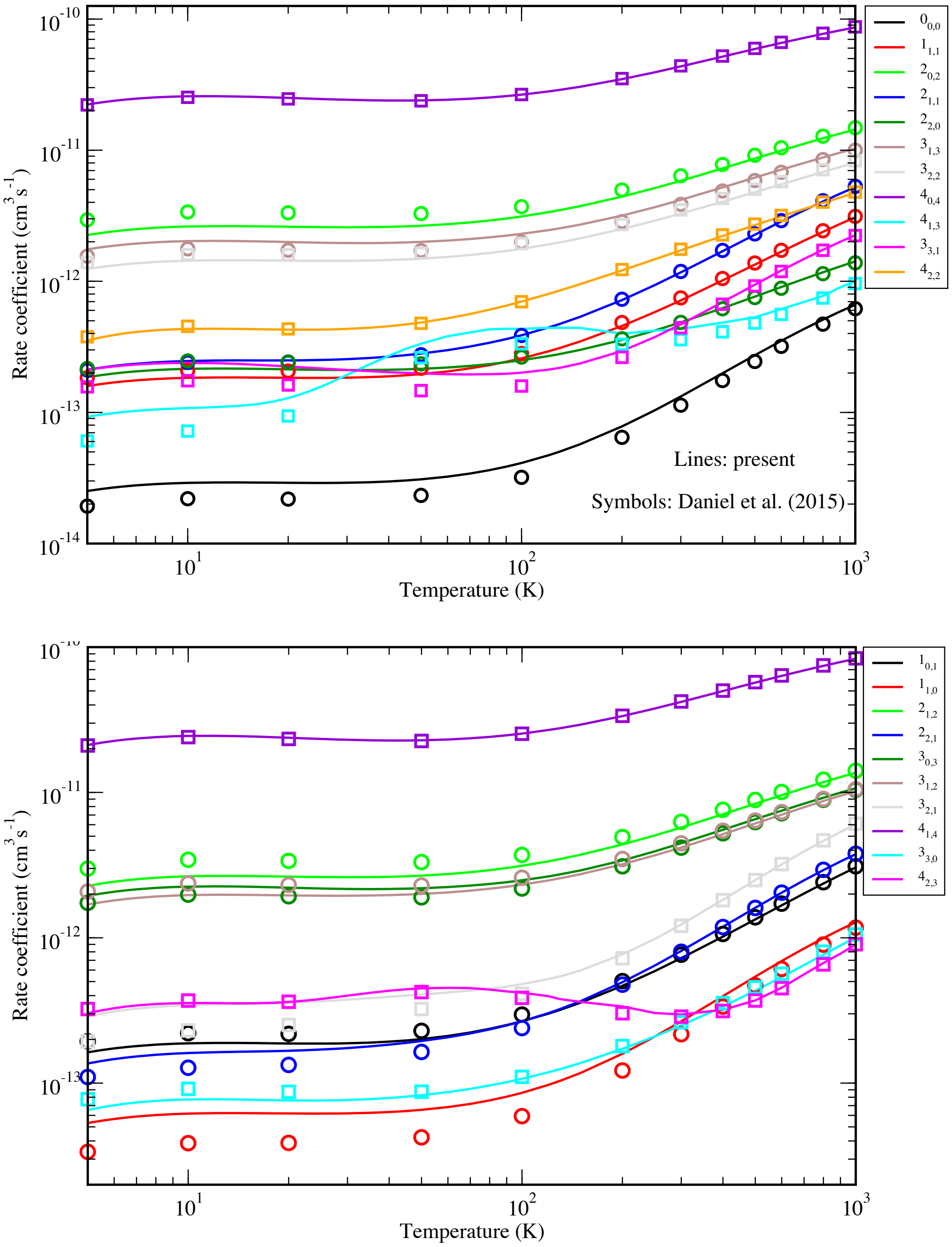

Figure S2: Rotational state-to-state de-excitation rate coefficients of H_2O in collision with H. Upper: para-H_2O from initial state $5_{1,5}$. Final states are: $0_{0,0}$, $1_{1,1}$, $2_{0,2}$, $2_{1,1}$, $2_{2,0}$, $3_{1,3}$, $3_{2,2}$, $4_{0,4}$, $4_{1,3}$, $3_{3,1}$, $4_{2,2}$. Lower: ortho-H_2O from initial state $5_{0,5}$. Final states are: $1_{0,1}$, $1_{1,0}$, $2_{1,2}$, $2_{2,1}$, $3_{0,3}$, $3_{1,2}$, $3_{2,1}$, $4_{1,4}$, $3_{3,0}$, $4_{2,3}$.

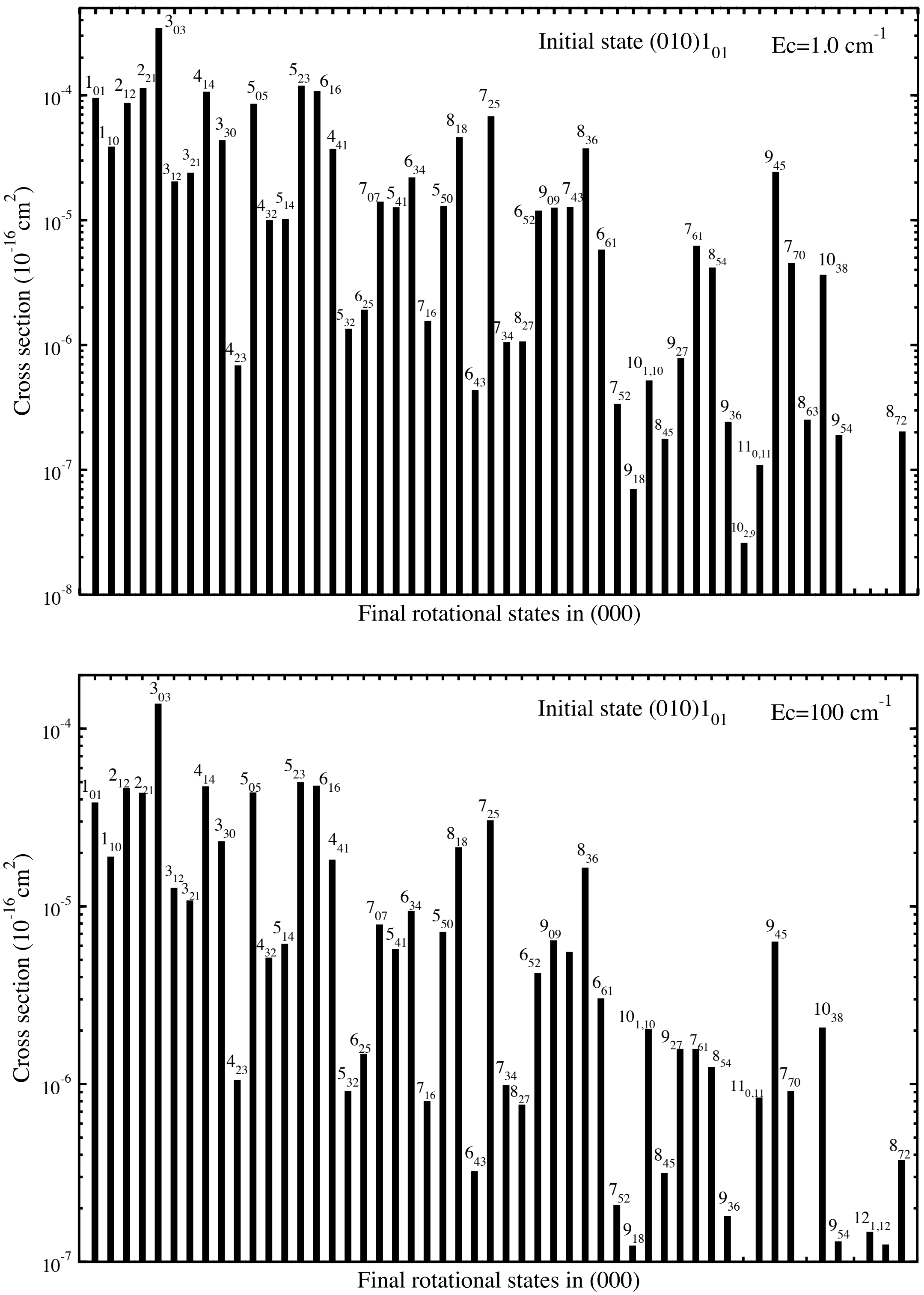

Figure S3: Rotational state distribution of ortho-H_2O in (000) from initial state $(010)1_{01}$ in collision with H. Left: collision energy = 1.0 cm^{-1}; Right: collision energy = 100.0 cm^{-1}.

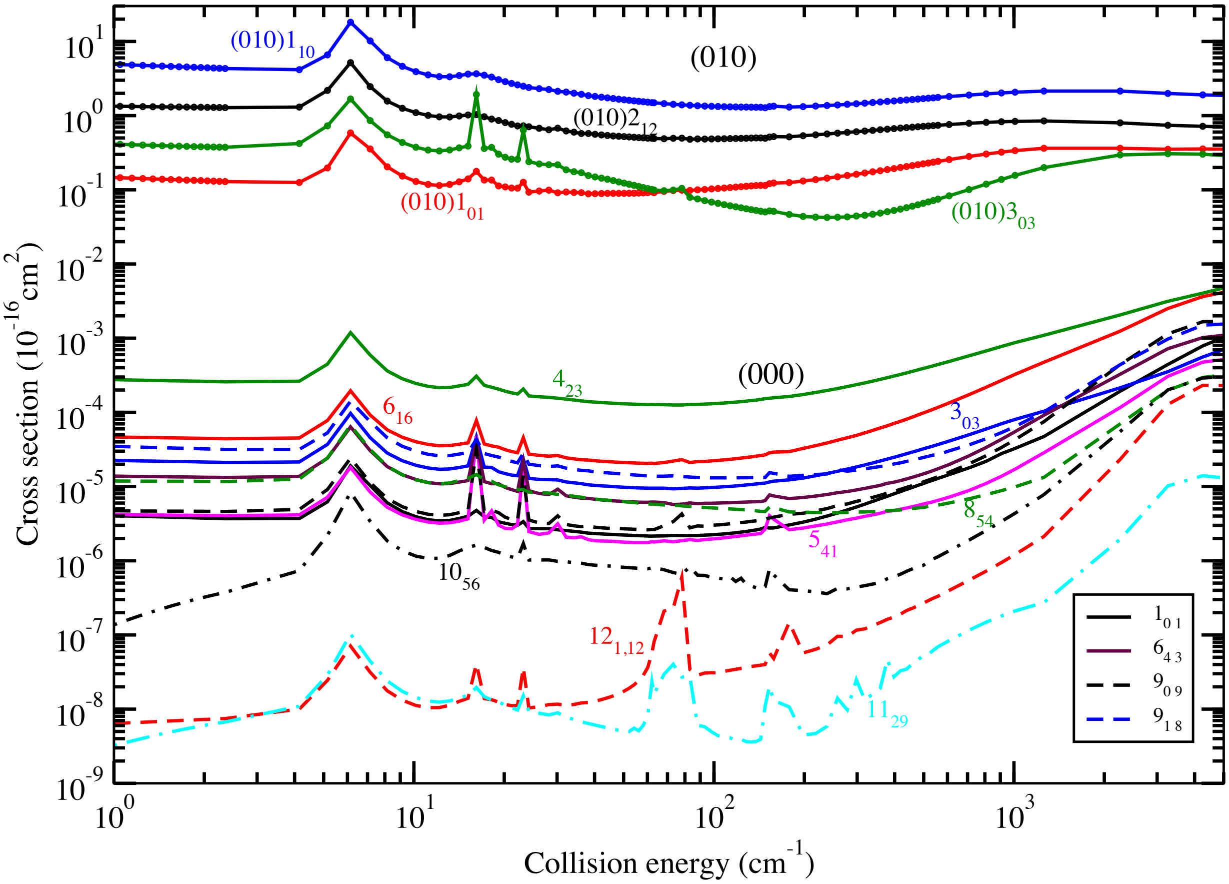

Figure S4: State-to-state rovibrational quenching cross sections of H_2O in collision with H from initial ortho-H_2O state $(010)2_{21}$. Final states are indicated on the curves.

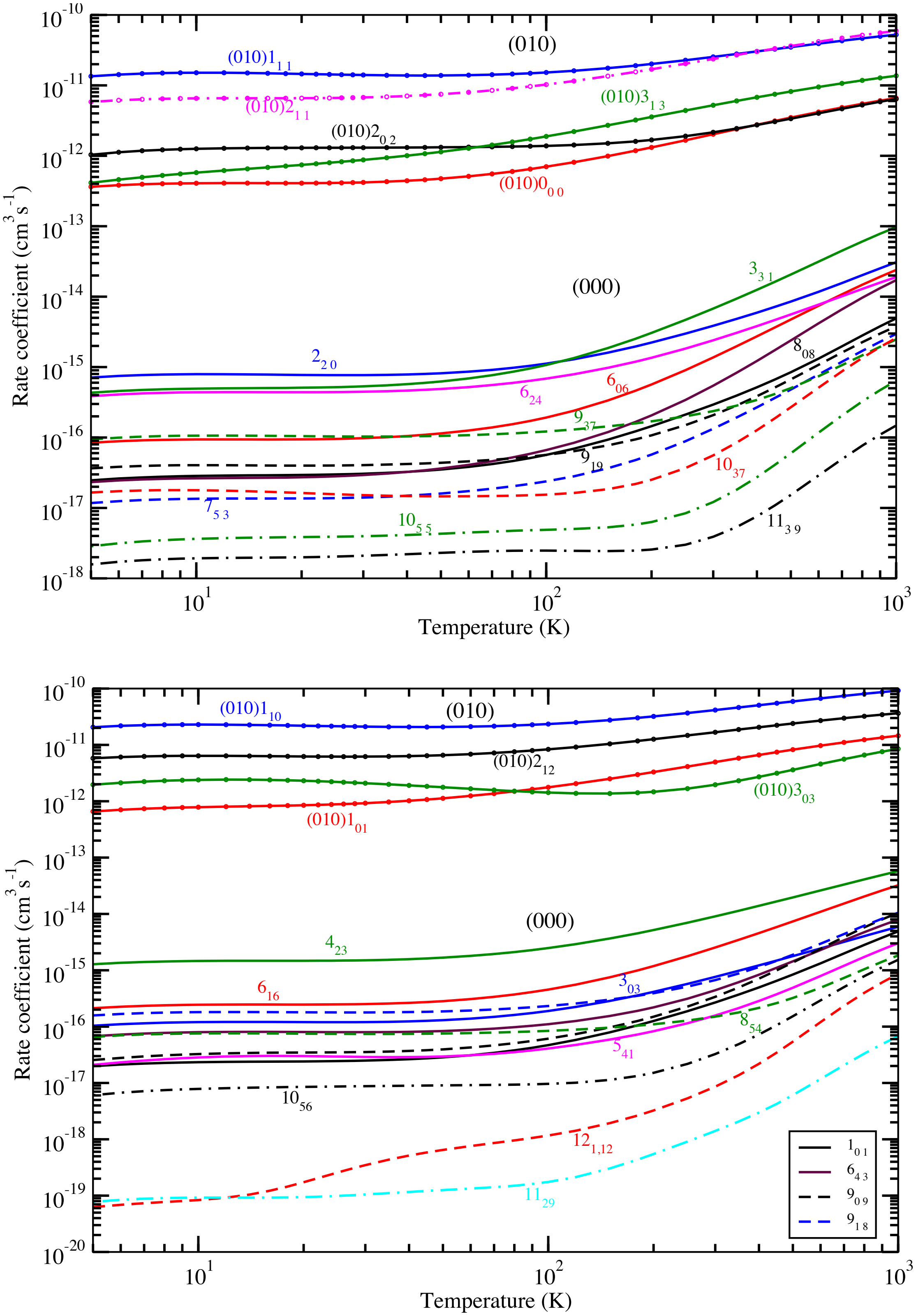

Figure S5: State-to-state rovibrational quenching rate coefficients of H_2O in collision with H. Upper: from initial para-H_2O state $(010)2_{20}$; Lower: from initial ortho-H_2O state $(010)2_{21}$. Final states are indicated on the curves.

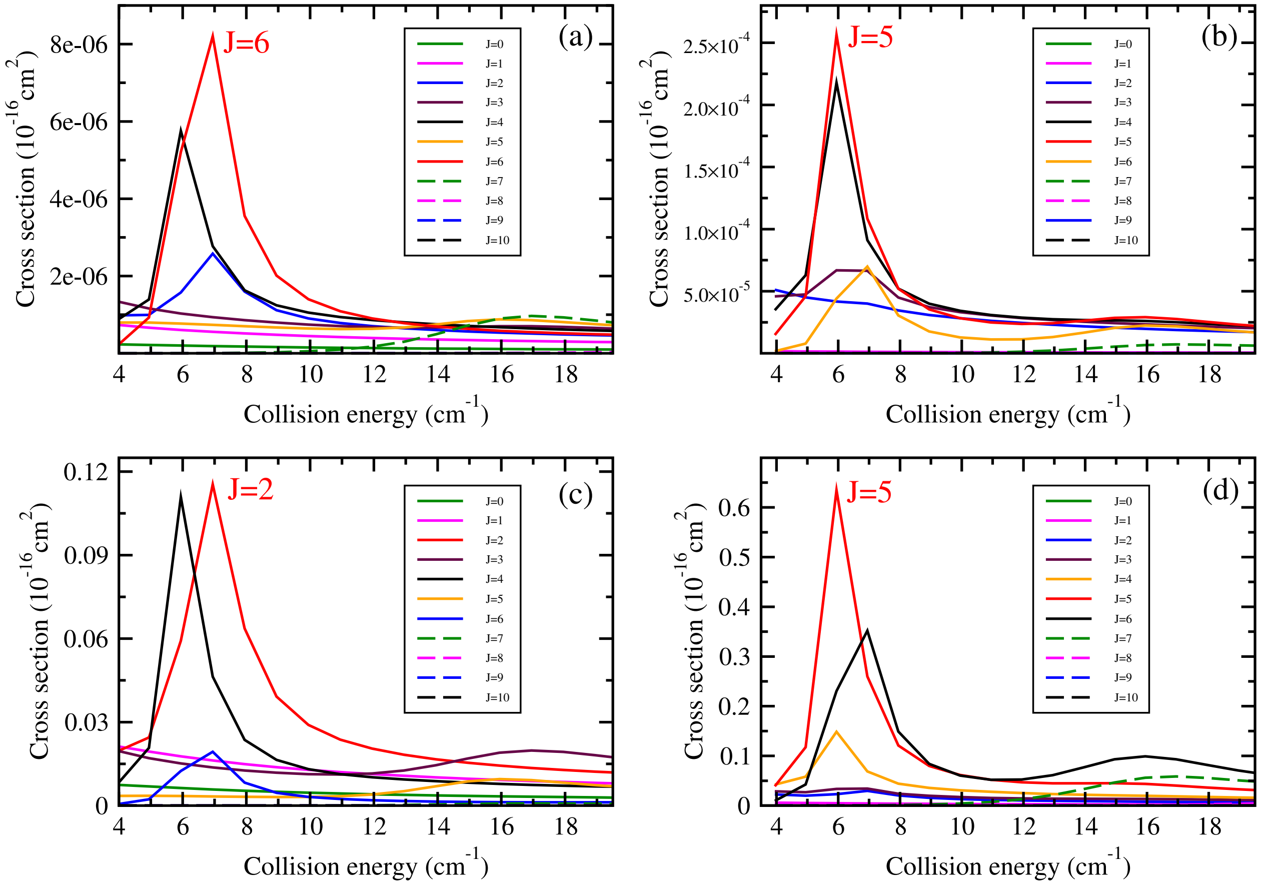

Figure S6: The J-resolved quenching cross sections as functions of collision energy from initial state $(010)2_{20}$. The final states are: (a) $(000)0_{00}$, (b) $(000)2_{20}$, (c) $(010)0_{00}$, and (d) $(010)2_{02}$.

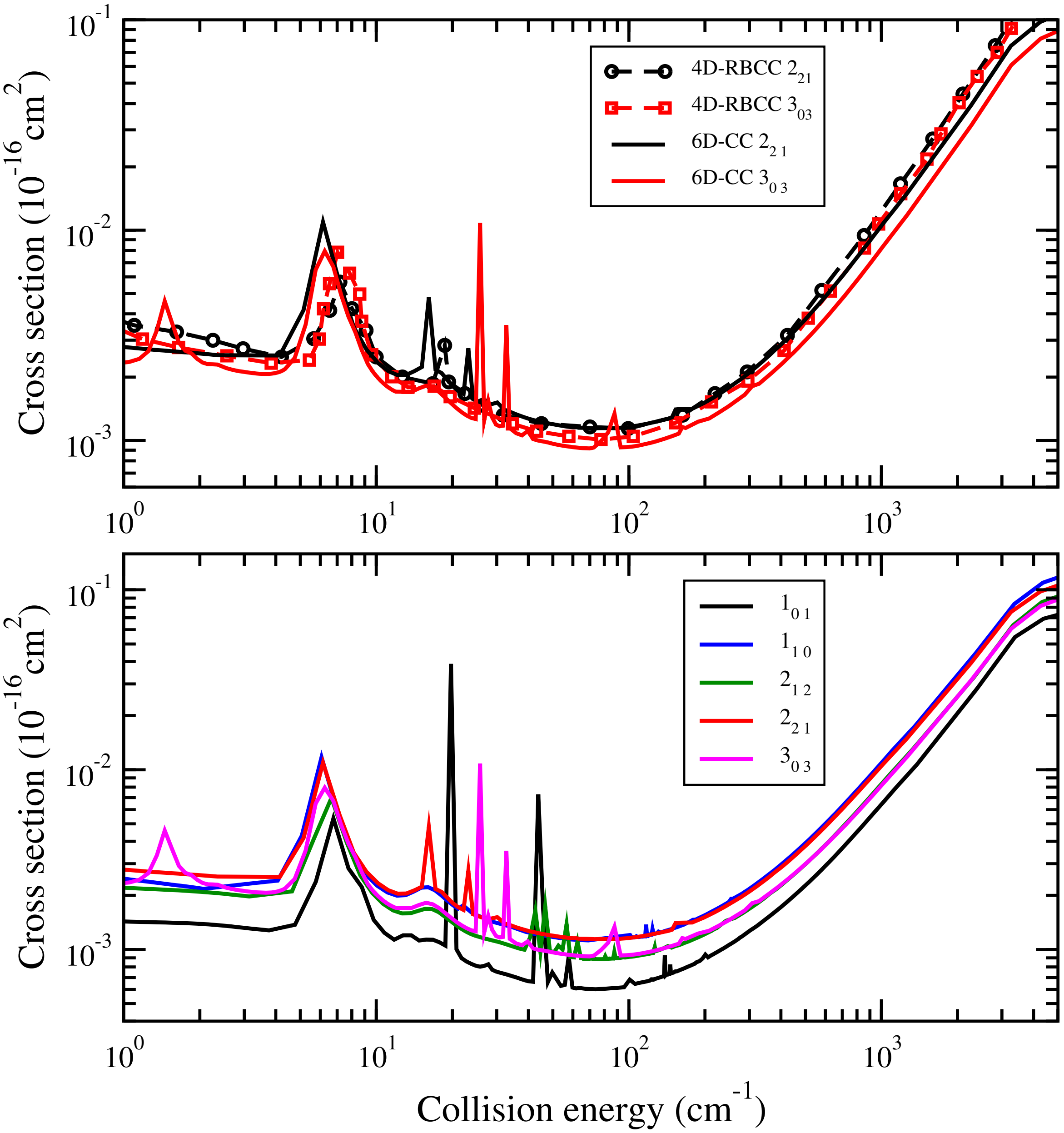

Figure S7: Total quenching cross section from $(010)j_{K_a,K_c}$ to (000) of ortho-H_2O in collision with H. (Upper) Comparison between present 6D-CC calculation with the 4D-RBCC results of Cabrera-González et al.[1] from initial state $(010)2_{21}$ and $(010)3_{03}$. (Lower) Present results from initial state $j_{K_a,K_c} = 1_{01}$, 1_{10}, 2_{12}, 2_{21}, and 3_{03}.
