# Supplementary material for: Inelastic Triatom-Atom Quantum Close-Coupling Dynamics in Full Dimensionality: All Rovibrational Mode Quenching of Water Due to the H Impact on a Six-Dimensional Potential Energy Surface
Source: J Phys Chem Lett. 2024 Nov 4;15(45):11312–9. doi: 10.1021/acs.jpclett.4c02865 (PMC11571227; doi:10.1021/acs.jpclett.4c02865)
Supplement: Supplementary file 2 — jz4c02865_si_002.pdf [file jz4c02865_si_002.pdf]

jz-2024-02865z.R1

Name: Peer Review Information for "Inelastic Triatom-Atom Quantum Close-Coupling Dynamics in Full Dimensionality: All Rovibrational Mode Quenching of Water Due to H Impact on a Six-Dimensional Potential Energy Surface"

First Round of Reviewer Comments

Reviewer: 1

Comments to the Author

This manuscript presents the calculation of cross sections for rovibrational transitions in the water molecule induced by collisions of an H atom. Water is an abundant molecule in many environments, and full interpretation of its spectroscopic observations requires collisional rate data.

The novel feature of this work is that all vibational degrees of freedom are included in the calculation. Previous work had considered de-excitation of the bending vibrational mode; this work presents the first calculations of de-excitation of the stretching degrees of freedom.

This reviewer has several questions/comments for the authors to deal with:

(1) Shouldn't the vibrational degrees of freedom as variables on which the potential V depends be included in Eq. 1? Similarly, h_{ABC} also should include a vibrational daependence.

(2) Given the large scale of these calculations, it would be helpful to the reader to have more information on the details of the scattering calculations, for instance the range of total angular momentum J required for converged calculation of the integral cross sections, the size of the scattering rovibrational basis (recognizing that the size depends on J), etc.

Typo: Acknowledgements, line 1: "support" -> "supported"

Reviewer: 2

Comments to the Author

A novel full six-dimensional quantum dynamics treatment of inelastic collisions of H with H_2O is presented. A new ab initio based 6D potential energy surface is computed and fitted. The inelastic quantum dynamics calculations were performed using the new full-dimensional coupled-channel ABC + D code. This work represents the first numerically exact full-dimensional treatment of the rovibrational quenching for all water vibrational modes with atomic hydrogen. Previous treatments used various approximations in reduced dimensions. Accurate inelastic data is crucial for the development of predictive astrophysics models and the results of this work will have significant and broad impact.

Minor suggestions to consider:

1) The authors should add the units of the contours to the Fig 1 caption.

2) The "O" is missing in H_2O in the first sentence of the second paragraph below Eq. S.1.

3) A prominent resonance feature is seen in nearly all of the cross sections for collision energies near 5 to 8 cm^-1. It would be interesting if the authors could add a sentence or two commenting on the origin of this resonance? Perhaps, a time-delay analysis would identify the channels/vibrational modes involved?

Author's Response to Peer Review Comments:

October 27, 2024

Editorial Office
The Journal of Physical Chemistry Letters

Dear Editor,

Please find enclosed our submission of the revised paper:

Manuscript ID: jz-2024-02865z

Inelastic Triatom-Atom Quantum Close-Coupling Dynamics in Full Dimensionality: all rovibrational mode quenching of water due to H impact on a six-dimensional potential energy surface

by **Benhui Yang, Chen Qu, J. M. Bowman, Dongzheng Yang, Hua Guo, N. Balakrishnan, R. C. Forrey, and P. C. Stancil**.

We thank the reviewers for their careful reading of the manuscript and for the very useful comments. We have attempted to address the comments of the reviewers and provide below a list of changes made to the manuscript and responses to the reviewers. All changes throughout the revised main manuscript and supporting information are highlighted in red.

Sincerely,

Benhui Yang et al.

encl: Responses to the reviewers' comments

Response to Reviewer: 1

This manuscript presents the calculation of cross sections for rovibrational transitions in the water molecule induced by collisions of an H atom. Water is an abundant molecule in many environments, and full interpretation of its spectroscopic observations requires collisional rate data.

The novel feature of this work is that all vibational degrees of freedom are included in the calculation. Previous work had considered de-excitation of the bending vibrational mode; this work presents the first calculations of de-excitation of the stretching degrees of freedom.

This reviewer has several questions/comments for the authors to deal with:

1. (1) Shouldn't the vibrational degrees of freedom as variables on which the potential V depends be included in Eq. 1? Similarly, h_{ABC} also should include a vibrational daependence.

   Author reply: In Eq. 1, the interaction potential V is expressed as a function of $(R, r_1, r_2, \theta_1, \theta_2, \phi)$, here the Radau coordinates r_1, r_2 and θ_1 are used to describe the vibrational modes of H_2O.

   We rewrote Eq. 1 to show the coordinate (r_1, r_2, and θ_1) dependence in the Hamiltonian for ABC, $\hat{h}_{ABC}$.

2. (2) Given the large scale of these calculations, it would be helpful to the reader to have more information on the details of the scattering calculations, for instance the range of total angular momentum J required for converged calculation of the integral cross sections, the size of the scattering rovibrational basis (recognizing that the size depends on J), etc.

   Author reply: To ensure the convergence of cross sections, a sufficient number of partial waves (J) and rovibrational basis were used in our scattering calculations. At the bottom of page 8, we added the discussion of these convergence tests.

3. Typo: Acknowledgements, line 1: "support" − > "supported" Author reply: The typo in Acknowledgements is corrected.

Response to Reviewer: 2

A novel full six-dimensional quantum dynamics treatment of inelastic collisions of H with H2O is presented. A new ab initio based 6D potential energy surface is computed and fitted. The inelastic quantum dynamics calculations were performed using the new full-dimensional coupled-channel ABC + D code. This work represents the first numerically exact full- dimensional treatment of the rovibrational quenching for all water vibrational modes with atomic hydrogen. Previous treatments used various approximations in reduced dimensions. Accurate inelastic data is crucial for the development of predictive astrophysics models and the results of this work will have significant and broad impact.

Minor suggestions to consider:

1. 1) The authors should add the units of the contours to the Fig 1 caption.

   Author reply: One sentence "The unit of potential energy is cm^{-1}." is added in the figure caption of Fig. 1.

2. 2) The "O" is missing in H2O in the first sentence of the second paragraph below Eq. S.1.

   Author reply: We corrected the typo.

3. 3) A prominent resonance feature is seen in nearly all of the cross sections for collision energies near 5 to 8 cm^{-1}. It would be interesting if the authors could add a sentence or two commenting on the origin of this resonance? Perhaps, a time-delay analysis would identify the channels/vibrational modes involved?

   Author reply: We added discussion about the resonances in the first paragraph of page 10 of main manuscript.

   We added Fig. S6 in the supporting information for the *J*-resolved quenching cross sections as functions of collision energy from initial state $(010)2_{20}$.
   Fig. S6 was discussed in the first paragraph of page S4.

   We identified the partial wave contribution to the resonances at collision energies near 5 to 8 cm^{-1}, and show in Fig. S6 the *J*-resolved contributions to the quenching cross sections from initial state $(010)2_{20}$ to final states $(000)0_{00}$, $(000)2_{20}$, $(010)0_{00}$, and $(010)2_{02}$.
